# The Relationship between Oscillations in Brain Regions and Functional Connectivity: A Critical Analysis with the Aid of Neural Mass Models

**DOI:** 10.3390/brainsci11040487

**Published:** 2021-04-12

**Authors:** Giulia Ricci, Elisa Magosso, Mauro Ursino

**Affiliations:** Department of Electrical, Electronic and Information Engineering, Campus of Cesena, University of Bologna, 47521 Cesena, Italy; giulia.ricci29@unibo.it (G.R.); elisa.magosso@unibo.it (E.M.)

**Keywords:** cortical rhythms, connectivity, neural mass models, excitatory and inhibitory synapses, Granger causality, nonlinear neural phenomena

## Abstract

Propagation of brain rhythms among cortical regions is a relevant aspect of cognitive neuroscience, which is often investigated using functional connectivity (FC) estimation techniques. The aim of this work is to assess the relationship between rhythm propagation, FC and brain functioning using data generated from neural mass models of connected Regions of Interest (ROIs). We simulated networks of four interconnected ROIs, each with a different intrinsic rhythm (in θ, α, β and γ ranges). Connectivity was estimated using eight estimators and the relationship between structural connectivity and FC was assessed as a function of the connectivity strength and of the inputs to the ROIs. Results show that the Granger estimation provides the best accuracy, with a good capacity to evaluate the connectivity strength. However, the estimated values strongly depend on the input to the ROIs and hence on nonlinear phenomena. When a population works in the linear region, its capacity to transmit a rhythm increases drastically. Conversely, when it saturates, oscillatory activity becomes strongly affected by rhythms incoming from other regions. Changes in functional connectivity do not always reflect a physical change in the synapses. A unique connectivity network can propagate rhythms in very different ways depending on the specific working conditions.

## 1. Introduction

Brain functioning depends on the interaction among different regions, which exchange information via a complex connectivity network and work together in a coordinated manner to realize cognitive tasks. Accordingly, the study of brain connectivity has been receiving increasing attention in cognitive neuroscience, as documented by the large amount of research in the field (see, among the others [1,2,3,4]). Indeed, there is large consensus that connectivity is a primary means for understanding brain function at different levels of organization. In fact, connectivity analysis has been assessed noninvasively in several recent studies, both starting from data obtained with magnetic resonance [5,6,7] or via neuroelectric imaging techniques (MEG or EEG) [8,9,10]; this analysis is of great value to encompass the relationships among the different areas involved, to unmask their specific role, and, ultimately, to understand how these interactions produce cognition in a coordinated fashion. In humans, invasive connectivity studies can also be performed with electrocorticography (ECoG) (i.e., placing electrodes at the brain surface [11], for instance, in the presurgical evaluation of epilepsy or during deep-brain stimulation therapy. The significance of brain connectivity estimates, however, is still the subject of large debate in the literature. A traditional distinction (which, however, has been questioned recently; see [1]) discerns between functional connectivity (FC) and effective connectivity estimates. According to a traditional point of view, the first assesses “the statistical dependence or mutual information between two neuronal systems” [2], whereas the second represents the causal influence that one neural system exerts on another, based on an explicit model describing the underlying process. Both, however, are different from the structural connectivity, defined as the presence of a physical connection among the regions. 

The previous definitions suffer from a variety of problems and are often misunderstood. In the following, we will especially refer to methods for the estimation of functional connectivity, but within the wider point of view proposed by Reid et al. [1] recently. These authors clearly underlined that, although methods for FC estimation are based on the computation of some forms of statistical or information association between signals, the target is always to understand the causal interactions among the different neural populations. Hence, the ultimate objective is to construct a causal network in order to comprehend how populations interact causally to produce cognition, and how alterations in these connections affect behavior (for instance, in pathological states). In other terms, the objective is to gain a mechanistic insight into brain functioning via a distributed process of structurally connected neural groups, although these networks are derived from associations among signals.

As underlined by Reid et al. [1], within this larger framework an essential role is now played by methods to validate FC estimation techniques against a series of ground-truth conditions. A typical way to implement validation is through the use of neurocomputational models inspired by brain functioning. Typically, simulated signals can be used to test whether methods for FC estimation are able to identify the structural connectivity imposed on the model, and, via a sensitivity analysis, to detect changes induced by parameter manipulation in the theoretical network.

Indeed, several such studies have been published in the last two decades, providing important confirmations but also widening the debate. Within these studies, it is possible to identify two main categories of employed models: those which simulate individual spiking neurons and ionic channels, mainly oriented to the study of connectivity in neuron cultures, and more abstract models, which employ neurons with continuous outputs and are more concerned with the study of connectivity among entire brain regions.

In the following, we will focus attention on a particular kind of abstract model—Neural Mass Models (NMMs) (for a summary on some connectivity studies with spiking neurons, see Ursino et al. 2020 [12], and also [13]). Rather than modeling all individual neurons within a brain circuit, NMMs simulate averaged activity generated by a population of similar neurons.

We decided to focus on NMMs since they represent a good compromise between accuracy and simplicity. They allow oscillatory phenomena to be described in a clear mechanistic way (emphasizing, for instance, the role of the different subpopulations involved) whilst still maintaining a limited number of state variables. It is easier to understand the behavior of such models compared with detailed models with spiking neurons, and it is easier to perform a sensitivity analysis across a wide range of conditions (as carried out in the present work). Parameters have a more general meaning, and so it is easier to generalize the validation results over different conditions. Of course, these models also have some limits. They cannot be used to incorporate results taken from individual neurons (for instance, to simulate the effect of ionic channels or drugs as measured directly on individual neurons) and their dynamics may exhibit some differences compared with the exact dynamics resulting from large populations of spiking neurons.

David et al. [14] tested the capacity of some FC estimators (cross-correlation, mutual information, and synchronization indices) to detect changes in neural coupling in a symmetric configuration of two NMMs: each measure was found to be sensitive to variations in neuronal coupling, with a monotonic dependence between the functional connectivity measures and the coupling parameter. Ansari-Asl et al. [15] and Wendling et al. [16] employed various NMMs (but with only two populations each) connected with an excitatory coupling parameter and explored the relationship between the coupling parameter and various FC estimates. They suggested that there are no ideal methods and that it is strongly advised to compare the outcomes from different connectivity estimates. Wang et al. [3] performed a systematic analysis on the performance of 42 different FC estimators against five different generations models (including convolution NMMs) with a five-node connectivity structure. Their results suggest that, when using signals generated with NMMs, Granger causality and Transfer Entropy (TE) show the ability to retrieve the underlying model structure quite well. However, difficulty can be encountered when the generative models include stronger nonlinearities (such as Rossler and Henon equations). However, in the study by Wang et al., only the classification accuracy was tested, using Receiver Operating Characteristic (ROC) curves, without an analysis on the relationship between the connectivity strength and the FC metrics.

All previous studies provide important indications on the virtues and limitations of several FC estimators, but also exhibit several important limitations. First, they did not test networks whilst including inhibitory couplings. Nevertheless, inhibition plays an important role in brain functioning, both to avoid excessive uncontrolled excitation spreading in the brain, and to implement competition mechanisms among different brain regions. Second, the previous studies did not test the effect of nonlinear behavior carefully. We claim that the estimate of a connectivity network via FC methods is strongly affected by the specific functioning of the different neural units involved—above all by their working point in the nonlinear neuron characteristics. Third, brain rhythms (in the α, β, γ and θ frequency ranges) are known to play a fundamental and specific role in many cognitive tasks (such as working memory, episodic memory, internal and external attention, perceptual grouping [17,18,19,20]). In particular, the idea that different brain regions are characterized by different intrinsic rhythms, and that these rhythms are transmitted from one region to another to produce a sophisticate “system of rhythms”, subserving various cognitive functions, has received important support in recent papers [21,22,23,24,25,26] and plays a fundamental role in neuroscience (see the final section of this work for a discussion on this aspect). Unfortunately, the problem of how these rhythms can propagate within a structural connectivity network and modulate their power has not been investigated in previous FC studies, despite its enormous relevance for the present cognitive neuroscience research. 

In a recent work [12], we investigated the capacity of an important FC estimator (the bivariate transfer entropy [27,28]) to detect changes in connectivity using signals generated by NMMs of interconnected ROIs (with two to four coupled regions). The connectivity network included excitatory and inhibitory links, and simulations were performed both in linear conditions and altering the working point of the individual regions. We found that TE can consistently estimate the strength of connectivity if neural populations work in their linear regions, and if the signal lengths are longer than 10 s. However, nonlinear phenomena strongly alter the TE estimation results; indeed, TE describes the amount of information transferred from one region to another, which is different from a true causal relationship. In that work, however, all regions were characterized by populations with identical internal parameters, producing a similar rhythm typically in the β band (which is fundamental to study activity in supplementary motor-premotor-primary motor cortical areas [29]). Hence, the previous study did not address the problem of how different rhythms (in different frequency bands) can be transmitted within a causal network and whether this rhythm propagation can be detected via common FC estimation metrics. 

The aim of the present paper is to significantly improve the previous research by analyzing the capacity of different and frequently used FC metrics to evaluate rhythm propagation and causal connectivity in a network of four interconnected ROIs, assuming that each ROI is characterized by a specific rhythm (in the bands θ, α, β or γ, respectively). Specifically, we tested eight different bivariate FC metrics, either nondirected (correlation, phase synchrony, coherence, lagged coherence) or directed (delayed correlation, temporal Granger causality, frequency Granger causality, transfer entropy). Moreover, while five of the previous metrics provide just a single value, three of them (coherence, lagged coherence and frequency Granger causality) provide a frequency-dependent estimation, allowing a discrimination among different frequency bands.

The paper is structured as follows. First, the NMM is qualitatively described, assigning parameters to each ROI to simulate the four different rhythms. Then, the eight methods used to assess FC are briefly described. The performances of these metrics are compared using 100 networks of connectivity among the four ROIs, generated randomly and including excitatory and inhibitory connections. Finally, a more complete analysis is performed using signals generated through a physiologically inspired connectivity network, simulating the interaction among rhythms in the occipital, parietal and frontal regions. In the main text, this analysis is performed using the Granger estimators, and concerns both changes in connectivity strength and alterations in the network working point. Results of additional simulations (performed with all estimators and on a different network) are presented in the Supplementary Materials, together with NMM mathematical equations. 

## 2. Method

In the following, the neural mass model is first described qualitatively. Then, we present the eight FC estimators used in the present work.

### 2.1. Neural Mass Model

**Figure 1:**
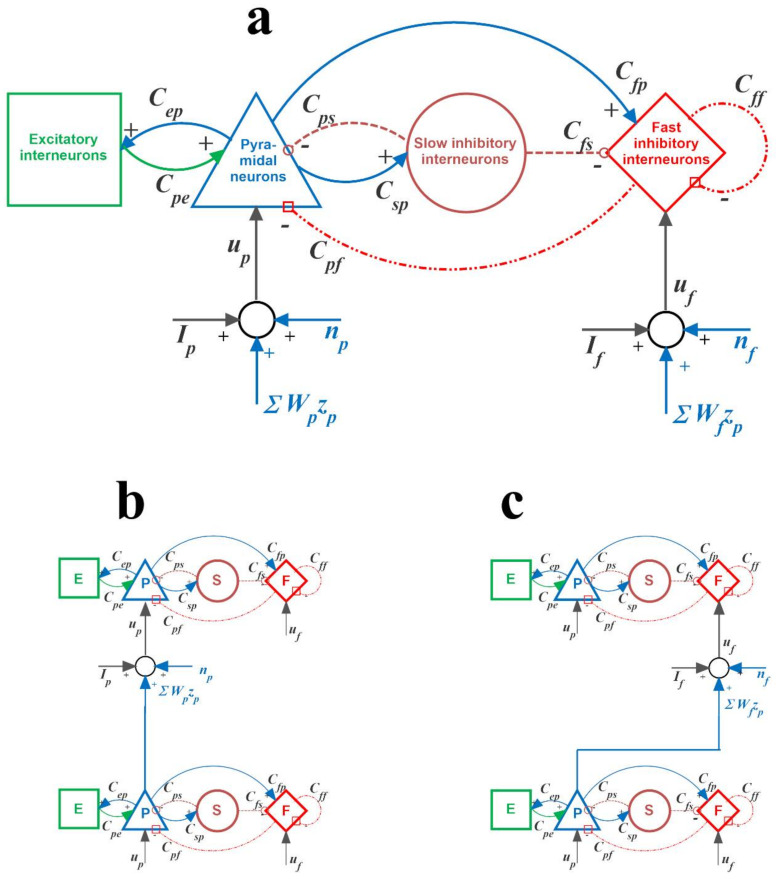
(**a**) Scheme of the neural mass model simulating the dynamics in a single Region of Interest (ROI). Blue continuous lines with arrows indicate glutamatergic excitatory synapses; red dash-dotted lines with open squares indicate GABAergic faster inhibitory synapses; brown dotted lines with open circles indicate GABAergic slower inhibitory synapses. Symbol *C_ij_* denotes the synaptic contacts among the neural populations where the first subscript and the second subscript represent the postsynaptic population and presynaptic population, respectively. *u_p_* and *u_f_* represent inputs to the pyramidal neuron population and to the fast inhibitory interneuron population, respectively. These inputs can come from the external environment (*I_p_* and *I_f_*, respectively), from noise (*n_p_* and *n_f_*, respectively) or from glutamatergic synapses from pyramidal neurons in other ROIs. (**b**) an example of excitatory connections between two ROIs via a direct link from the pyramidal neurons of the source ROI to the pyramidal neurons of the target ROI. (**c**) an example of a bisynaptic inhibitory connection, from the pyramidal neurons of the source ROI to the fast inhibitory neurons of the target ROI (which, in turn, inhibit pyramidal neurons in the target ROI).

*Model of a single ROI:* A single Region of Interest (ROI) was simulated via the feedback arrangement among four neural populations: pyramidal neurons (subscript *p*), excitatory interneurons (subscript *e*), and inhibitory interneurons with slow and fast synaptic kinetics (GABA_A,slow_ and GABA_A,fast_, subscripts *s* and *f*, respectively). This structure simulates the fundamental excitatory and inhibitory connections occurring within a cortical column, although in a drastically simplified manner. Each population receives an average postsynaptic membrane potential (say *v*) from other neural populations (according to the schema depicted in Figure 1), and converts it into an average firing rate (say *z*). To account for the nonlinear phenomena in neural dynamics (lower threshold and upper saturation), this conversion was simulated with a static sigmoidal relationship.

We used a second-order system to simulate synaptic kinetics in Figure 1. However, we distinguished three types of synapses, assigning different values to the parameters (gain and time constant) of the second-order transfer function: (i) glutamatergic *excitatory* synapses, including both synapses from pyramidal neurons and from excitatory interneurons (assuming they have similar dynamics, with gain *G_e_* and time constant *1/ω_e_*); (ii) GABAergic *inhibitory* synapses with *slow* dynamics (parameters *G_s_* and *1/ω_s_*); (iii) GABAergic *inhibitory* synapses with *fast* dynamics (parameters *G_f_* and *1/ω_f_*). Overall, the synaptic connections among the neural populations within a ROI are represented by eight parameters C_ij_, with the first and second subscripts representing the postsynaptic and presynaptic populations, respectively.

As shown in the results section, by assigning proper values to the internal connectivity parameters and synaptic time constants in each ROI, we realized four different regions with different intrinsic rhythms (in the θ, α, β and γ ranges, respectively). These four ROIs could then be connected in various feedback networks, according to the long-range connectivity patterns described below.

*Connections among ROIs*: According to present neurophysiological knowledge, a network of interconnected ROIs can be realized assuming that long-range synapses emerge only from pyramidal neurons of the source ROI (hence, they are glutamatergic in type with impulse responses *h_e_*(*t*)). At least in principle, these long-range connections can target any population, either inhibitory or excitatory of the target ROI. For the sake of simplicity, however, in the present work, as in previous ones [30,31,32], we assumed that excitatory long-range connections among ROIs can target only postsynaptic pyramidal neurons or inhibitory interneurons with fast GABAergic dynamics. In the first case, the source ROI exerts an excitatory influence on pyramidal neurons in the target ROI via a direct monosynaptic connection. In the second case, the influence is inhibitory via a bisynaptic connection; specifically, the first is a long-range excitatory connection directed from the pyramidal neurons in the source ROI to the inhibitory interneurons in the target ROI, while the second is a local inhibitory connection from the interneurons to the pyramidal neurons within the target ROI. Exempla are illustrated in panels B and C of Figure 1.

As illustrated in Figure 1, each ROI, in addition to receiving long-range synapses from other ROIs, may receive inputs from the external environment (*I_p_* and *I_f_*, entering into the pyramidal population and fast inhibitory population, respectively) and superimposed Gaussian white noise. Here, we assumed that only the external input *I_p_* to the pyramidal population is non-null in the four simulated ROIs, while *I_f_* is kept at 0 in each ROI.

However, it is worth noting that in our model the random noise is not applied directly to the LFP of pyramidal neurons (i.e., the quantity *v_p_* in Equation (4) of the Supplementary Materials part 1), but it acts on pyramidal neurons through the typical low-pass dynamics of the glutamatergic synapses (this is described in Equations (5) and (6) of the Supplementary Materials part 1). Hence, the effective noise on *v_p_* exhibits a low-pass shape that resembles the true 1/f noise of LFPs (see Figure S1 in the Supplementary Materials part 2). Of course, it may be of value, in future work, to directly use realistic noise extracted from real LFP recordings, which may contain other frequencies coming from different inputs. This may significantly contribute to making the model spectra more reliable.

All model equations can be found in the Supplementary Materials part 1.

In the present work, to assess the performance of the eight different FC estimators, we first generated 100 random networks connecting the four ROIs, with the same external input *I_p_* (=400) to each ROI. We assumed that each network can have a number of long-range synapses ranging between 3 and 9 (randomly chosen, the others are set a zero) with connection strengths as great as 10, 20, 30, and 40 (randomly chosen). Each connection can be either excitatory or inhibitory, with 50% probability of each.

**Figure 2:**
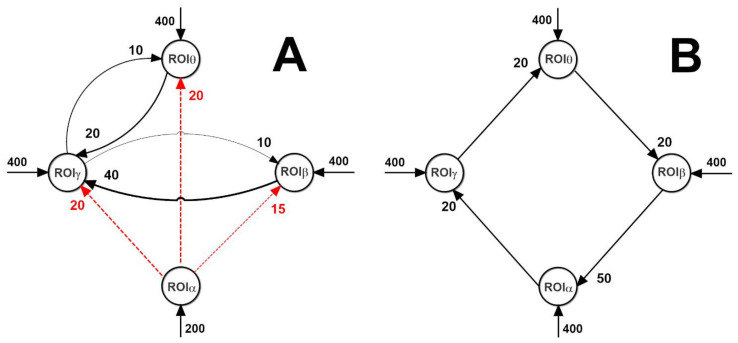
Connectivity networks among the four ROIs used in the present work (dashed red lines and continuous black lines denote inhibitory and excitatory connections, respectively). The network in (**A**) simulates a possible physiological connectivity from occipital (or thalamic) regions to motor regions and temporal/frontal regions. This network, by varying the strength of a single connection or the input value to a single ROI, was used to obtain the results shown in Figures 6–13 using the functional connectivity (FC) metrics based on Granger causality (but see also Figure S4 in Supplementary Materials part 2 for the other FC metrics). The simple loop network in (**B**) allows a straightforward analysis of rhythm propagation in a chain of interconnected ROIs; the corresponding results are reported in Supplementary Materials part 2 (Figures S5 and S6).

Subsequently, we tested the FC estimators on the two connectivity networks depicted in Figure 2 by varying both the connection strength and the inputs to the ROIs (the latter changes have been performed to test nonlinear effects). Only results on the first network (in Figure 2A) are illustrated in the main text. All other results are summarized in the Supplementary Materials part 2.

Most of the results shown in the present work consider networks with a similar number of excitatory and inhibitory (bisynaptic) connections. Indeed, several authors stress that the excitatory and inhibitory ensembles are well balanced in the human cortex and that a breakdown of this balance may contribute to several pathological states, such as epilepsy [33]. However, we also tested other networks similar to those used in this study, but with all pyramidal–pyramidal synapses; the main results did not change meaningfully.

For each generated network, and for each combination of inputs to the network, ten different simulations were performed using random noise superimposed on the inputs (normal distribution with zero mean value and SD = $\sqrt{5/dt},$ where *dt* is the simulation step). Any group of ten simulations was repeated starting from the same seed to be sure that differences can be ascribed only to the network connections and to the input values, rather than to the particular noise. In the following, the result of each FC estimate is the average of the values obtained in the ten trials.

The set of differential equations (see Supplementary Material part 1) was numerically integrated with the Euler method, with an integration step as low as 10^−4^ s. The duration of each simulation was 11 s, but the first second was excluded from the subsequent computations to avoid the confounding effects of the initial transient phenomena. As discussed in Ursino et al. [12], a 10 s length for the signals ensures a good reliability of the estimates.

The FC estimates were performed using the simulated membrane potentials of pyramidal neurons (quantity *v_p_*, see Supplementary Materials part 1), as a good approximation of EEG or of mean field potentials for each ROI. To reduce the computational cost, simulated signals were resampled at 100 Hz after low pass-filtering with an antialiasing zero-phase filter (cut-off frequency 50 Hz).

### 2.2. Functional Connectivity Estimates

We used eight different bivariate methods to estimate FC. Let us assume that the presynaptic and postsynaptic signals are described by two discrete stochastic processes (say ***x***[*n*] and ***y***[*n*], respectively, where we use the boldface to denote a random variable). In the following, we will use *x*[*n*] and *y*[*n*] (without bold) to represent two particular realizations of the stochastic processes, where *n* is the discrete time (*n* = 0, 1, …, *N* − 1).

*Pearson correlation coefficient*: the expression of this is:

(1) $$r_{yx}=\frac{\sum_{i=0}^{N-1}\left(x\left[i\right]-\bar{x}\right)\left(y\left[i\right]-\bar{y}\right)}{\sqrt{\sum_{i=0}^{N-1}{\left(x\left[i\right]-\bar{x}\right)}^{2}\sum_{i=0}^{N-1}{\left(y\left[i\right]-\bar{y}\right)}^{2}}}$$

where $\bar{x}$ and $\bar{y}$ represent the average values of the corresponding quantity. Of course, this estimator is nondirected (i.e., *r_yx_* = *r_xy_*). Moreover, it can be positive or negative to discriminate between excitatory or inhibitory connections.

*Delayed correlation coefficient*: this coefficient differs from the previous one since the postsynaptic signal is delayed, assuming that a finite time is necessary to propagate information from *x* to *y*. Hence, we can write:

(2) $$dr_{yx}=\underset{d}{\max}\left|\frac{\sum_{i=0}^{N-d-1}\left(x\left[i\right]-\bar{x}\right)\left(y\left[i+d\right]-\bar{y}\right)}{\sqrt{\sum_{i=0}^{N-d-1}{\left(x\left[i\right]-\bar{x}\right)}^{2}\sum_{i=0}^{N-d-1}{\left(y\left[i+d\right]-\bar{y}\right)}^{2}}}\right|$$

where *d* is the delay (expressed as the number of samples); hence, if ∆*t* is the sampling period, the overall temporal delay is *d*·∆*t*. It is worth noting that, as in many other studies, *d* was chosen as the value that maximizes the absolute value in Expression (2). However, the correlation coefficient can assume a positive or negative value. Hence, we used the absolute value to choose the value of *d* for a given connection, and to compute true and false positives in ROC curves. Conversely, we maintained the sign (positive or negative) in other figures to investigate whether this metrics can detect the presence of excitatory or inhibitory connections.

*Coherence*: this estimator has been computed as the magnitude squared coherence function

(3) $$C_{yx}\left(f\right)=\frac{{\left|P_{yx}\left(f\right)\right|}^{2}}{P_{xx}\left(f\right)P_{yy}\left(f\right)}$$

where *f* is frequency, $P_{yx}\left(f\right)$ is the cross-spectral density of the two signals, $P_{xx}\left(f\right)$ is the power spectral density of *x* and $P_{yy}\left(f\right)$ is the power spectral density of *y*. All power densities were computed using the Welch periodogram method [34], with a 0.5 s window (50 samples) and 10 s zero padding (1000 samples) to ensure a spectral resolution as sharp as 0.1 Hz. Coherence, of course, is a nondirected estimator ($C_{yx}\left(f\right)=C_{xy}\left(f\right)$) and provides an estimate at each frequency of the discrete power spectra.

*Lagged coherence*: a possible limitation in the use of coherence is that it is affected by zero-lag (instantaneous) correlations, which can artificially inflate the estimated values. Among the measures proposed to mitigate this issue, the lagged coherence was developed by Pascual-Marqui et al. [35] to detect physiological lagged connections between brain regions and is not affected by volume conduction and by low spatial resolution. It is defined as follows

(4) $$LC_{yx}\left(f\right)=\frac{{\left(Im\left\{P_{yx}\left(f\right)\right\}\right)}^{2}}{P_{xx}\left(f\right)P_{yy}\left(f\right)-{\left(Re\left\{P_{yx}\left(f\right)\right\}\right)}^{2}}$$

where $Im\left\{\cdot \right\}$ and $Re\left\{\cdot \right\}$ denote the imagery and real part of the corresponding complex-valued argument and the remaining symbols on the right have the same meaning as in Equation (3). Lagged coherence is a nondirected connectivity measure ($LC_{yx}\left(f\right)=LC_{xy}\left(f\right)$).

*Phase synchronization*: to estimate phase synchronization (see [16,36]), we first computed the analytical signal

(5) $$Z_{x}\left[n\right]=x\left[n\right]-\bar{x}+jH\left[x\left[n\right]-\bar{x}\right]=A_{x}\left[n\right]e^{j\varphi_{x}\left[n\right]}$$

where H[.] denotes di Hilbert transform and $j=\sqrt{-1}$. It is worth noting that we subtracted the average value of the signal before computing the analytical form. Of course, a similar expression holds for the *y* signal too. Then, we computed the phase difference between the two signals at any discrete time *n*:

(6) $$∆\varphi_{yx}\left[n\right]=\varphi_{y}\left[n\right]-\varphi_{x}\left[n\right]$$

Phase synchronization was finally obtained by estimating the quantity: $\left|E\left\{e^{j∆\varphi_{yx}}\right\}\right|$, where E{.} represents the statistical mean value. The latter has been estimated as follows

(7) $$PS_{yx}=\left|\frac{1}{N}\overset{N-1}{\underset{i=0}{\sum }}e^{j∆\varphi_{yx}\left[i\right]}\right|$$

which provides a scalar nondirected quantity $PS_{yx}=\;PS_{xy}$.

*Time-Domain Granger Causality*: this estimate is based on the autoregressive (AR) modeling framework and compares the prediction ability of two AR models of the same process ***y***[*n*]—i.e., a univariate AR model and a bivariate AR model; in the latter, the current value of the process ***y***[*n*] was predicted not only based on its past values (as in the univariate case), but also on the past values of the other process ***x***[*n*]. Specifically, we can write

(8) $$y\left[n\right]=\overset{p}{\underset{k=1}{\sum }}a\left[k\right]y\left[n-k\right]+\eta_{y}\left[n\right]$$

(9) $$y\left[n\right]=\overset{p}{\underset{k=1}{\sum }}b\left[k\right]y\left[n-k\right]+\overset{p}{\underset{k=1}{\sum }}c\left[k\right]x\left[n-k\right]+\varepsilon_{y}\left[n\right]$$

for the univariate and bivariate AR model, respectively, where *p* is the order of the model. $\eta_{y}\left[n\right]$ and $\varepsilon_{y}\left[n\right]$ are white noise processes and represent the model’s residual (or prediction error) in each case. The variance of the residual (let us say γ and $\sigma_{yy}$, respectively) quantifies the quality of the model fit. The Granger causality from *x* to *y* in the time domain is defined as [37,38]:

(10) $$GC_{yx}=\ln\frac{var\left(\eta_{y}\left[n\right]\right)}{var\left(\varepsilon_{y}\left[n\right]\right)}=\ln\frac{\gamma }{{\;\sigma }_{yy}\;}$$

where, of course, in practice the variances will be estimated on the particular realizations of the residuals. A substantial reduction in the variance of the residual in case of the bivariate compared to univariate model means that including the past values of *x* provides a better prediction model for *y*, and $GC_{yx}$ is substantially larger than 0—i.e., *x* casually influences *y* in the Granger sense. Similarly, Granger causality from *y* to *x*, $GC_{xy}$, was computed via the same procedure, building the AR models for the process ***x***[*n*]. Granger causality is a directed connectivity estimator $\left(GC_{yx}\neq GC_{xy}\right)$.

*Frequency-domain (spectral) Granger causality:* Granger causality can be formalized in the spectral domain [38,39] starting from the joint bivariate autoregressive representations of the two processes:

(11) $$\overset{p}{\underset{k=0}{\sum }}A\left[k\right]\left[\begin{array}{c} x\left[n-k\right]\\ y\left[n-k\right] \end{array}\right]=\left[\begin{array}{c} \varepsilon_{x}\left[n\right]\\ \varepsilon_{y}\left[n\right] \end{array}\right]$$

Equation (11) is derived from Equation (9) and the analog one expressing the bivariate model of $x\left[n\right]$; $A\left[k\right]$ are 2 × 2 coefficient matrices (identity matrix at time lag 0). After Fourier transforming Equation (11), we manipulated it to obtain

(12) $$\left[\begin{array}{c} X\left(f\right)\\ Y\left(f\right) \end{array}\right]=\left[\begin{array}{cc} H_{xx}\left(f\right) & H_{xy}\left(f\right)\\ H_{yx}\left(f\right) & H_{yy}\left(f\right) \end{array}\right]\left[\begin{array}{c} {Ε}_{x}\left(f\right)\\ {Ε}_{y}\left(f\right) \end{array}\right]=H\left(f\right)\left[\begin{array}{c} {Ε}_{x}\left(f\right)\\ {Ε}_{y}\left(f\right) \end{array}\right]\;H\left(f\right)=A^{-1}\left(f\right)$$

This is the transfer function matrix. By right multiplying each side of Equation (12) by its conjugate transpose (*), the cross-spectral density matrix $S\left(f\right)$ for signals *x* and *y* can be expressed as

(13) $$S\left(f\right)=\;H\left(f\right)\Sigma H{\left(f\right)}^{*}$$

where $\Sigma =\left[\begin{array}{cc} \sigma_{xx} & \sigma_{xy}\\ \sigma_{xy} & \sigma_{yy} \end{array}\right]$ is the covariance matrix of the residuals (white noise processes) in Equation (11). The spectral Granger causality from *x* to *y* is computed as (for further mathematical details see [38])

(14) $$sGC_{yx}\left(f\right)=\ln\frac{P_{yy}\left(f\right)}{P_{yy}\left(f\right)-\left(\sigma_{xx}-\frac{\sigma_{xy}}{\sigma_{yy}}\right){\left|H_{yx}\left(f\right)\right|}^{2}}\;\;\;\;=\ln\frac{P_{yy}\left(f\right)}{P_{yy}\left(f\right)-\tilde{\sigma_{xx}}{\left|H_{yx}\left(f\right)\right|}^{2}}$$

The numerator expresses the total power spectrum of *y* at frequency f, while the denominator is the difference between the total power spectrum and the “causal” power exerted by signal *x* on signal *y* at the same frequency. Accordingly, the quantity $sGC_{yx}$ at a given frequency *f* is zero when the causal power of *x* onto *y* at *f* is zero and increases (>0) as the causal power increases. The spectral Granger causality from *y* to *x*, $sGC_{xy}\left(f\right)$, was obtained from Equation (14) by exchanging the subscripts *y* and *x*. Of course, this connectivity measure is directional ($sGC_{yx}\neq sGC_{xy}$).

*Transfer Entropy:* to calculate the transfer entropy from *x* to *y*, one first needs to construct the embedded vectors.

$$X^{m}\left[n\right]=\left[x\left(n\right)\;x\left(n-∆n\right)\;x\left(n-2∆n\right)\ldots \;x\left(n-\left(m-1\right)∆n\right)\right]\;Y^{h}\left[n\right]=\left[y\left(n\right)\;y\left(n-∆n\right)\;y\left(n-2∆n\right)\ldots \;y\left(n-\left(h-1\right)∆n\right)\right]$$

In previous equations, *m* and *h* are the embedding dimensions, defining the number of past samples used, and Δ*n* represents the embedding delay. These parameters serve to approximately reconstruct the state spaces of the pair of time series. Each vector, $X^{m}\left[n\right]$ and $Y^{h}\left[n\right],$ comprises the present and *m* − 1 (or *h* − 1) past samples of the particular realization of the random process.

The concept behind TE is that, in case of a causal influence from *x* to *y*, the probability of **y**[n], conditioned by its past $Y^{h}\left[n-∆n\right]$ only, should be lower than the probability of **y**[n] conditioned by both its past $Y^{h}\left[n-∆n\right]$ and the past of the other signal $X^{m}\left[n-∆n\right]$. This concept can be formalized by computing the corresponding reduction in entropy as the Kullback–Leibler divergence between the two probability distributions [40]. However, as discussed by Wibral et al. [41], the influence of a neural signal on another takes some time (e.g., tens of milliseconds) to be effective due to the traveling time of the action potential along the axons from the presynaptic to the postsynaptic region—that is, a pure delay (say *d*) in the neural interactions must be taken into account. If we assume that *d* can be approximated by *l* embedding delays (*d* = *l*⋅∆*n*), $X^{m}\left[n-∆n\right]$ can be replaced by the delayed signal $X^{m}\left[n-l∆n\right]$ in the definition of TE. In practice, *l* is generally unknown, and it needs to be estimated from the available data (see below). Based on this description, we acquired

(15) $$TE_{yx}=\underset{\begin{array}{c} y\left[n\right]\\ Y^{h}\left[n-∆n\right]\\ X^{m}\left[n-l∆n\right] \end{array}}{\sum }p\left(y\left[n\right],Y^{h}\left[n-∆n\right],X^{m}\left[n-l∆n\right]\right)log_{2}\frac{p\left(y\left[n\right]/Y^{h}\left[n-∆n\right],X^{m}\left[n-l∆n\right]\right)}{p\left(y\left[n\right]/Y^{h}\left[n-∆n\right]\right)}$$

Of course, transfer entropy is directional—i.e., $TE_{yx}\neq TE_{xy}$. A fundamental problem in the evaluation of Equation (15) is that various joint and marginal probability distributions (with very large dimensionality, up to *m* + *h* + 1) must be evaluated starting from the finite data samples. Moreover, several parameters (such as the embedding dimensions *m* and *h*, the embedding delay Δ*n* and the overall transmission delay, *l*Δ*n*) are unknown and require estimation from the data. In this study, we used the software package Trentool [28,42] to estimate TE from the outputs of the neural mass model. More details on the implementation can be found in our previous work [12].

Some of the metrics adopted to estimate functional connectivity are frequency-dependent (coherence, lagged coherence, spectral Granger causality); in order to derive a single value for each bivariate connection, the mean values of the estimated connectivity profile over the entire range of frequencies (4–40 Hz) was computed for each of these metrics.

## 3. Results

### 3.1. Power Density Spectra of the Different Regions

First, we assigned parameters to each ROI so that any region can produce an intrinsic rhythm in a different frequency band when excited in the central region of its sigmoidal relationship. Each ROI has been named considering its intrinsic rhythm. The considered bands are θ: 4–8 Hz (ROIθ), α: 8–13 Hz (ROIα), β: 13–26 Hz (ROIβ) and γ: 26–40 Hz (ROIγ). To this aim, we manually modified the parameters representing the synaptic contacts among the populations (*C_ij_*) and the reciprocal of time constants (*ω_j_*). All parameter values can be found in Supplementary Materials part 1.

**Figure 3:**
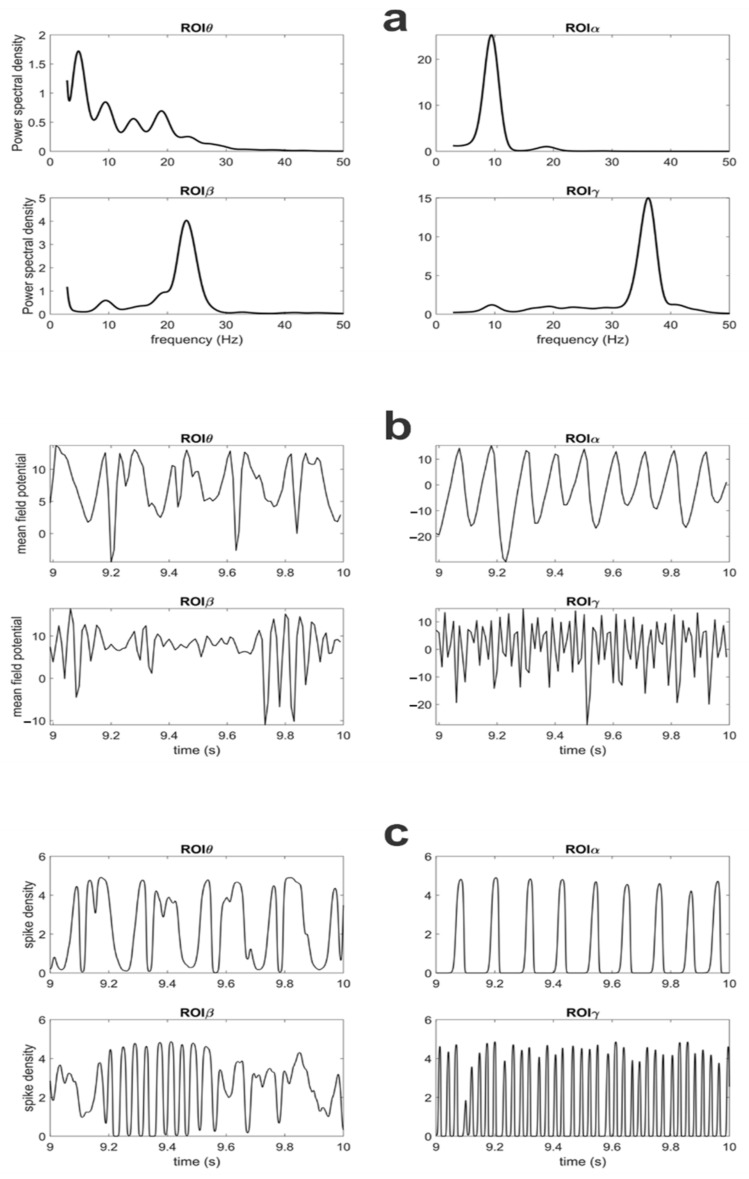
Power spectral densities of potential for the pyramidal population in the four different ROIs (**a**) simulated with the connectivity as in Figure 2A and all inputs as great as 400. Power spectral densities (PSDs) have been obtained by computing the Welch periodogram on membrane potentials of pyramidal neurons. Parameters for each ROI and noise levels are shown in Table S1 of Supplementary Materials part 1. (**b**,**c**) represent the temporal pattern of membrane potential of pyramidal neurons and their spike densities, respectively, during the last second of the simulation.

The power spectral densities (PSDs) of all ROIs and the temporal patterns of membrane potentials and spike density for pyramidal neurons, simulated with the connectivity as in Figure 2A, are illustrated in Figure 3 (see also Figure S2 in Supplementary Materials part 2, where spectrograms are shown).

The temporal patterns show that the rhythms in the model exhibit a complex waveform—i.e., they are not sinusoidal (as the patterns obtained, for instance, with the use of Wilson Cowan oscillators) and are characterized by intermittent fluctuations. As pointed out by Cole and Voytek [43] and by Jones [44], the use of complex intermittent waveforms is essential to reach a full comprehension of the role and meaning of brain rhythms.

### 3.2. Analysis of the Different Metrics with Random Network Connectivity

**Figure 4:**
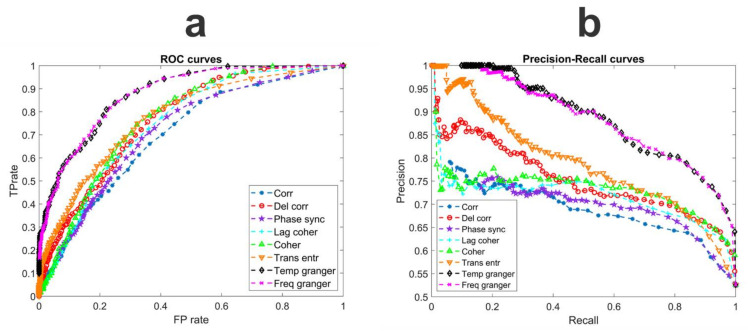
ROC curves (**a**) and precision–recall curves (**b**) obtained with the different FC estimators using the data obtained from 100 randomly generated connectivity networks among the four ROIs. The area under the ROC curve for each estimator is reported in Table 1.

In order to compare the performance of the different estimators, we generated 100 different random networks connecting the four ROIs (with a number of connections ranging between 3 and 9, synaptic weights of 10, 20, 30 or 40, equal probability of excitatory or inhibitory connections, and external input *I_p_* = 400 for each ROI; see Methods section). The performances were assessed by matching the connectivity network obtained via the estimator with the true connectivity network and quantifying this matching via ROC curves and precision–recall curves. This was performed on a binary basis (yes/no connection) by comparing the value estimated by each metrics with a threshold (range 0–0.5) and evaluating the percentage of true positives vs. the percentage of false positives at each threshold. The results are summarized in Figure 4. Finally, Table 1 reports the areas under the curves (AUCs) computed starting from the ROCs.

Results show that the Granger estimators (both in the temporal and frequency domains) provide a more reliable description of the connectivity network, with values of the AUCs as high as 0.88. TE and coherence also provide fair results (AUC = 0.77–0.78), whereas the performance of the other estimators is lower.

The precision–recall curves prove that our results are valid independently of a possible unbalance in the data set and further confirm that Granger and TE provide better results than the other estimators in the context of the present work. In particular, the precision (i.e., the capacity to reveal a connection only when it really exists) may be quite high with Granger and TE estimators using a high threshold; the delayed correlation exhibits a good precision too. Furthermore, even if the threshold is reduced, the Granger estimates maintain a good precision (higher than 80%), still maintaining a recall as high as 80% (i.e., by limiting the number of false negatives).

The previous analysis, however, is quite limited, since it just compares the topology of the true and estimated connectivity networks in terms of existence/nonexistence of a given connection. A more complete analysis requires the assessment of the connectivity weight and, even more importantly, the role of input changes and nonlinearity. This analysis will be shown in the next subsections, which use the Granger estimators, first on the random net and then on the network in Figure 2A. Results obtained with different estimators and on the net in Figure 2B are shown in Supplementary Materials part 2.

### 3.3. Analysis of the Connectivity Strength

**Figure 5:**
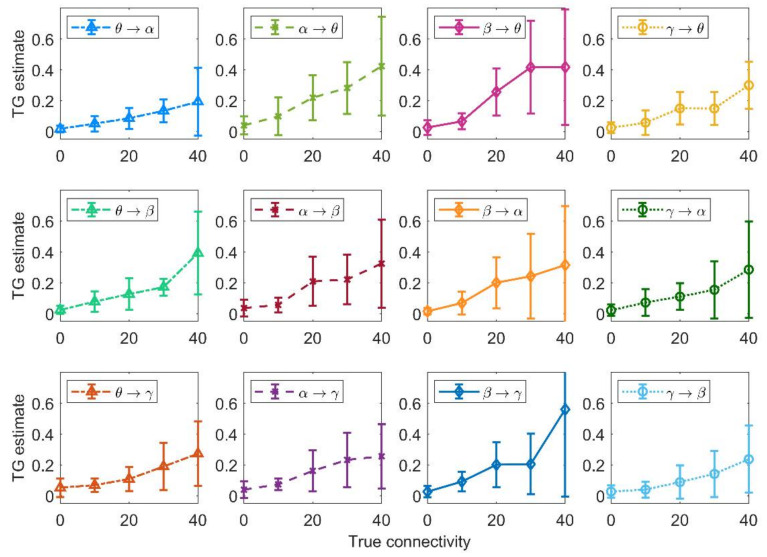
Relationship between the connectivity estimated with the temporal Granger causality and the true connectivity, obtained using the data from 100 randomly generated connectivity networks. It is worth noting that, in these nets, connections were randomly generated between 0 and 40 (step 10). Points are mean values at each connection strength, and bars denote standard deviations. The estimator is able to grasp the monotonic increase in connectivity. It is worth noting the large SDs occur especially when the connectivity is high and when a connection emerges from the ROIβ. See discussion.

In order to evaluate the capacity of the Granger estimator to detect changes in connectivity strength, we re-examined the results obtained with the 100 random networks generated above, plotting the connection values estimated with the temporal Granger estimator (mean ± SD) vs. the true value (Figure 5). All estimated values show a monotonic dependence on the true value, demonstrating that the estimator is able to catch the alterations in the connectivity strength even in a multivariate condition—i.e., when many connections vary together. On average, the estimator is able to distinguish the absence of a connection (0 value) from a moderate (10–20) or a higher (30–40) connection. However, the SD is quite high, especially at the highest values of the connection strength, indicating that a single estimated value is subject to large variability.

Moreover, from Figure 5 one can observe that the connection β → γ can produce higher values of FC than the other connections. Our impression is that the γ oscillations can be easily modulated by an incoming β rhythm, and this makes the connection β → γ particularly effective in influencing the dynamics of the target population.

The previous results show that, in a multivariate condition, just a tendency can be detected, but with a large SD. In the following, we will examine a different condition, i.e., the effect of a single change in connectivity strength, with all other connections maintained at a constant value. This can be of value to understand whether the Granger causality can detect a progressive alteration in one neural pathway (either due to learning or pathological conditions). This analysis was performed starting from the network depicted in panel A of Figure 2. Here, ROIα can represent either an occipital region (which is dominated by an intrinsic α rhythm) or the thalamus, which has been hypothesized to generate an α rhythm and transmit it to other populations [45,46]. The ROIβ can represent motor–premotor areas, where this rhythm becomes evident during motor activation or motor programming tasks [29]. Finally, the ROIγ and ROIθ can represent more fronto-temporal regions (for instance, those involved in working memory), where γ-θ coupling is known to play a pivotal role, or also γ-θ coupling may represent a connectivity between frontal regions and the hippocampus [19,47]. However, this network has been built just as a simple example inspired by present knowledge on brain rhythms. Results obtained with another net (depicted in Figure 2B) are shown in Supplementary Materials part 2 (Figures S5 and S6).

In the following, for briefness, connections among two ROIs are indicated by omitting the word ROI and leaving only the Greek symbols denoting the specific ROIs (e.g, θ → γ means connection from ROIθ to ROIγ—i.e., ROIθ→ROIγ).

**Figure 6:**
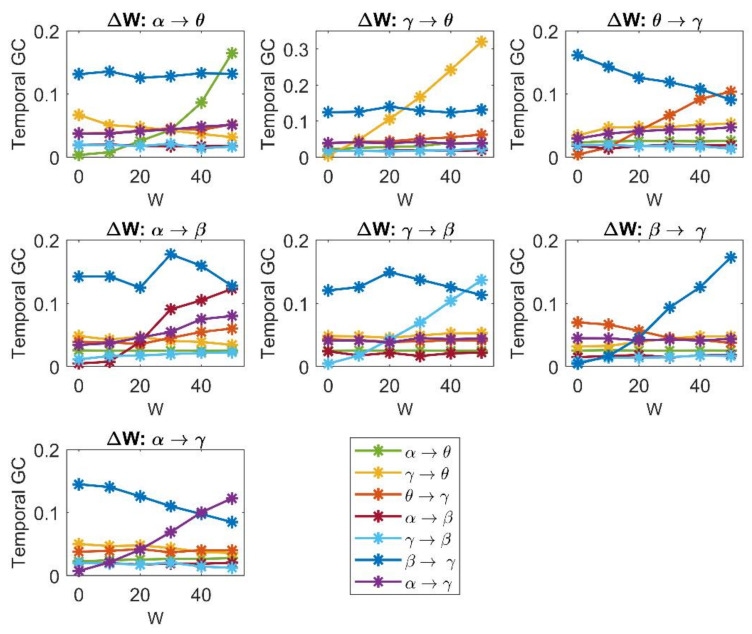
Values of connectivity among the ROIs estimated with the temporal Granger estimator, with reference to the network in Figure 2A, when one connection is progressively varied in the *x*-axis (from 0 to 50, with step of 10), and the other connections are maintained at the basal value as in Figure 2A. It is worth noting that the estimator is able to detect the progressive increase in a single synaptic strength, while the other estimates remain almost constant. As an exception, we observed that the increase in a synapse entering into ROIγ is often associated with a decrease in another synapse entering the same ROI. It is also worth noting the higher sensitivity of the estimator to the connection γ → θ.

Figure 6 shows the effect of a progressive change in a single connection strength on the temporal Granger estimates. To this end, each connection strength was individually increased from 0 to 50 (step 10), while all other connections were maintained at the basal value shown in Figure 2A; 10 simulations were performed and averaged for each configuration of the network. Several aspects are of interest.

First, in any case the Granger estimator is able to detect the change in a single connectivity quite well, with a monotonic and almost linear relationship between the estimated values and the connectivity strength. Only the θ → γ and α → β connections show a certain tendency to saturate at the upper values, while the α → θ connection shows a parabolic trend.

Second, in most cases the other estimates are unaffected by the change in one connection and remain quite constant at different values of the sensitivity parameter. We observed just a few exceptions, especially concerning the ROIγ: increases in a connection involving the γ population are associated with the “apparent” decrease in another connection targeting the same ROI (the increase in β → γ is associated with the “apparent” decrease in θ → γ; the increase in θ → γ with the “apparent” decrease in β → γ; the increase in α → γ with the apparent decrease in β → γ). These changes always concern a bisynaptic connections (such as β → γ → θ or α → β → γ). Furthermore, it is interesting to observe that a change in the connection α → β produces some effects also on the connections α → γ and θ → γ, which exhibit a moderate apparent increase. This is likely a consequence of the indirect effect of α on γ via β.

Third, the relationship between the “true” connectivity value and the estimated one exhibits quite a similar slope for all connections, with the exception of the connection γ → θ, which exhibit a higher slope than the others (see the different *y*-axis in this panel).

Moreover, it is worth noting that the slope of the relationship “estimated connectivity vs. true connectivity” is about half that illustrated in Figure 5. This may the consequence of the smaller input (=200) to the ROIα used in the network of Figure 2A (the network utilized for the analysis in Figure 6 and following figures) compared to the value used for this input (=400) in the analyses of Figure 4 and Figure 5 (but see also Figure S3 in Supplementary Materials part 2, where we compare the results obtained in the random net with two different inputs to ROIα, and further show how a change in the input affects connectivity).

The latter consideration moves our attention to the role of the inputs reaching the ROIs—i.e., a change in the population working point. This is assessed in the next subsection.

### 3.4. Effect of the Inputs on the Estimated Connective Strength

**Figure 7:**
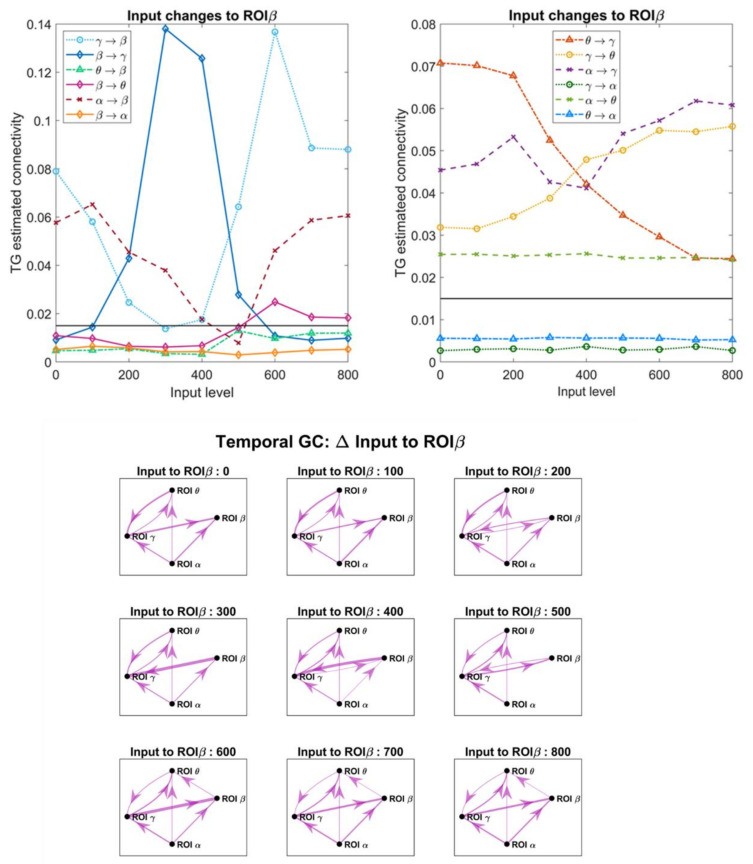
Upper panels: Values of connectivity among the ROIs estimated with the temporal Granger causality, with reference to the network in Figure 2A, when the input to pyramidal neurons in ROIβ was progressively varied from 0 to 800, as in the *x*-axis, and all other inputs and connections were maintained at the basal value, as in Figure 2A. It is worth noting the strong effect that the input change has on the connections which involve the ROIβ (in particular the output connection β → γ and the input connection γ → β). Additionally, the connection between ROIγ and ROIθ is affected. Bottom panels: connectivity graphs obtained from the estimates using a threshold as low as 0.015 (the threshold is depicted as a horizontal black line in the upper panels).

**Figure 8:**
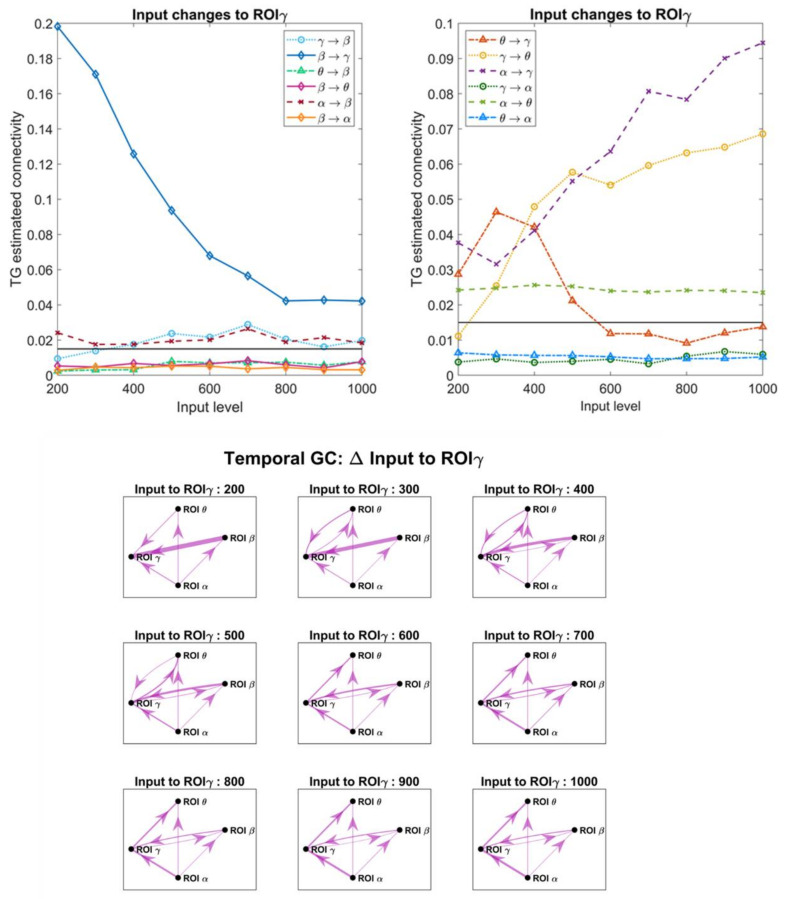
Upper panels: Values of connectivity among the ROIs estimated with the temporal Granger causality, with reference to the network in Figure 2A, when the *input* to pyramidal neurons in ROIγ was progressively varied from 200 to 1000, as in the *x*-axis, and all other inputs and connections were maintained at the basal value, as in Figure 2A. It is worth noting the strong effect that the input change has on several connections which involve the ROIγ. Bottom panels: connectivity graphs obtained from the estimates using a threshold as low as 0.015 (the threshold is depicted as a horizontal black line in the upper panels).

**Figure 9:**
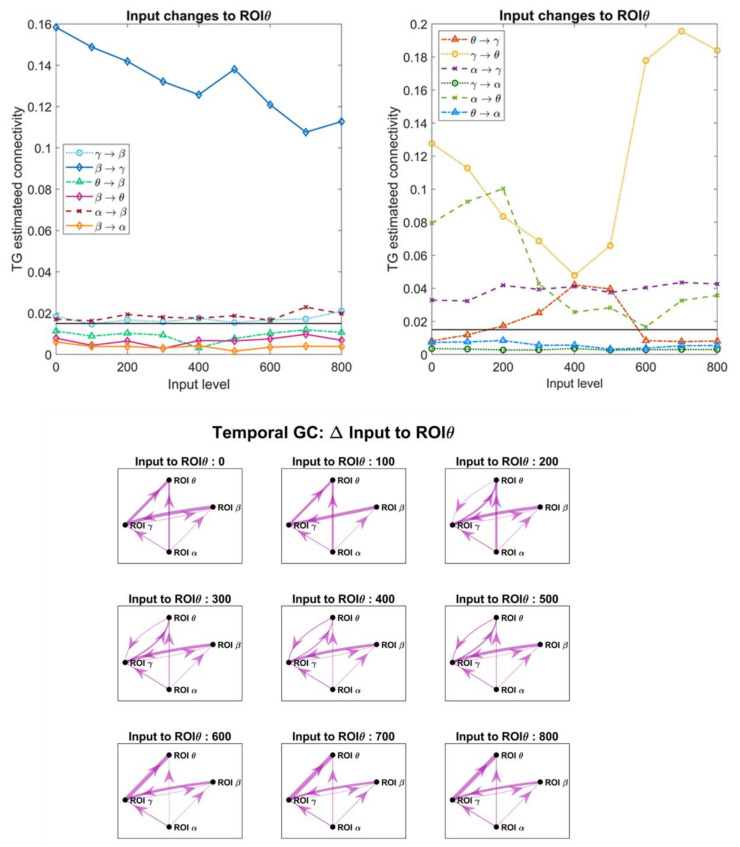
Upper panels: Values of connectivity among the ROIs estimated with the temporal Granger causality, with reference to the network in Figure 2A, when the *input* to pyramidal neurons in ROIθ was progressively varied from 0 to 800, as in the *x*-axis, and all other inputs and connections were maintained at the basal value, as in Figure 2A. It is worth noting the strong effect that the input change has on the connections which involve the ROIθ. Bottom panels: connectivity graphs obtained from the estimates using a threshold as low as 0.015 (the threshold is depicted as a horizontal black line in the upper panels).

**Figure 10:**
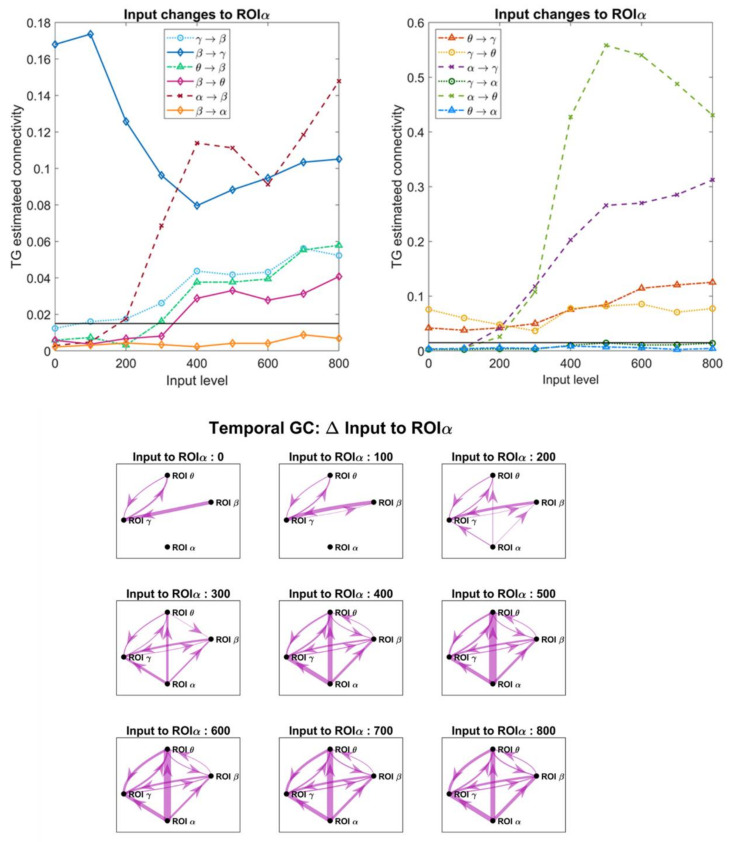
Upper panels: Values of connectivity among the ROIs estimated with the temporal Granger causality with reference to the network in Figure 2A, when the *input* to pyramidal neurons in ROIα was progressively varied from 0 to 800, as in the *x*-axis, and all other inputs and connections were maintained at the basal value, as in Figure 2A. It is worth noting that all estimated connections from ROIα to the other regions increase significantly. Many other connections increase too with the appearance of spurious terms. Bottom panels: connectivity graphs obtained from the estimates using a threshold as low as 0.015 (the threshold is depicted as a horizontal black line in the upper panels). The emergence of spurious connections is evident from these graphs.

In order to investigate the role of a change in the inputs, which in turn modifies the working point of a population along the sigmoidal relationship, we changed the excitation to pyramidal neurons in one ROI, while all other inputs were maintained at the value illustrated in Figure 2A. Figure 7, Figure 8, Figure 9 and Figure 10 display the effect of a change in the input to ROIβ, ROIγ, ROIθ and ROIα, on the estimates obtained with the temporal Granger causality. The upper panels show the values of each estimate as a function of the input, while the bottom panels show the corresponding patterns of connectivity, obtained using a threshold as high as 0.015 (taken as optimal from the ROC curve in Figure 4).

As is clear in Figure 7, Figure 8, Figure 9 and Figure 10, a change in the input has a significant effect on the estimated connections which enter into or exit from the given ROI. In particular:

(i). Changing the input to ROIβ (Figure 7) causes a dramatic change in the estimated connection β → γ, which exhibits a high value when the input to ROIβ is in the range 300–400, and falls to very low values when the input is 0–100 or 600–800. We ascribe this behavior to the fact that pyramidal neurons in the region ROIβ enters into the bottom or upper saturation zone of the sigmoidal relationship, hence providing a small output signal. Conversely, the entering connections γ → β and α → β exhibit the opposite behavior: they decrease in the central zone (input 300–400) and increase dramatically in the saturation regions. At the same time, the connections involving region ROIγ also change, as illustrated in the right panel of Figure 7.
(ii). Increasing the input to ROIγ (Figure 8) causes a progressive increase in the estimated output connection γ→θ and a dramatic fall in the entering connections β → γ and θ → γ.
(iii). Increasing the input to ROIθ (Figure 9) causes a significant change in the estimated output connection θ → γ. This connection is higher at intermediate levels of the input (300–500) and falls down when the region ROIθ enters into the bottom or upper saturation zones (input 0–100 or 600–800). The opposite pattern is evident as to the entering connection γ → θ, which increases when ROIθ is in the saturation zones and decreases in the central region. Additionally, the entering connection α → θ decreases in the central region but remains low also in the upper saturation region. This global behavior resembles that already described in Figure 7 when changing the input to ROIβ.
(iv). Increasing the input to ROIα (Figure 10) causes an evident increase in all the estimated output connections (α → β, α → γ and α → θ). This is paralleled by a progressive increase in most other connections, including the “spurious” connections β → θ and θ → β, which were set at zero in the original network. Using very high values for the inputs, the network overconnected.

The bottom panels in Figure 7, Figure 8, Figure 9 and Figure 10 show examples of connectivity networks, obtained using 0.015 as a threshold. As it is evident, some “true” connections can be lost or other “spurious” connections can emerge as a consequence of the input changes. In particular, in most figures the estimated connections not included in the network (that is, β → θ, θ → β, β → α, γ → α, θ → α) exhibit very low values below the discrimination threshold; however, when increasing the inputs, some of these connections can rise, causing false positive estimations.

Finally, Figure S4 in Supplementary Materials part 2 summarizes the effect of a change in the input to ROIβ (the same as in Figure 7) but evaluated with all estimators in order to better understand the differences between the different metrics. The main considerations developed above are confirmed, although with some differences among the different estimates.

### 3.5. Analysis in the Frequency Domain

**Figure 11:**
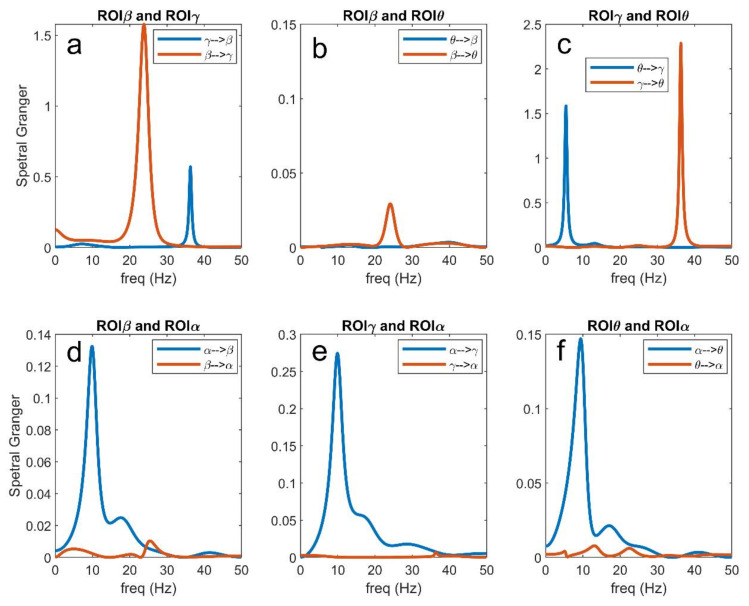
Values of connectivity among the ROIs estimated with the spectral Granger causality with reference to the network in Figure 2A and plotted as a function of frequency (note the use of different *y*-axes to emphasize the different cases). The estimator reproduced the network connectivity quite well: the exchange of rhythms between ROIβ and ROIγ (**a**) and between ROIγ and ROIθ (**c**) is evident; the coupling between ROIθ and ROIβ is negligible (**b**); and the α rhythm is clearly transmitted from ROIα to the other regions (**d**–**f**).

**Figure 12:**
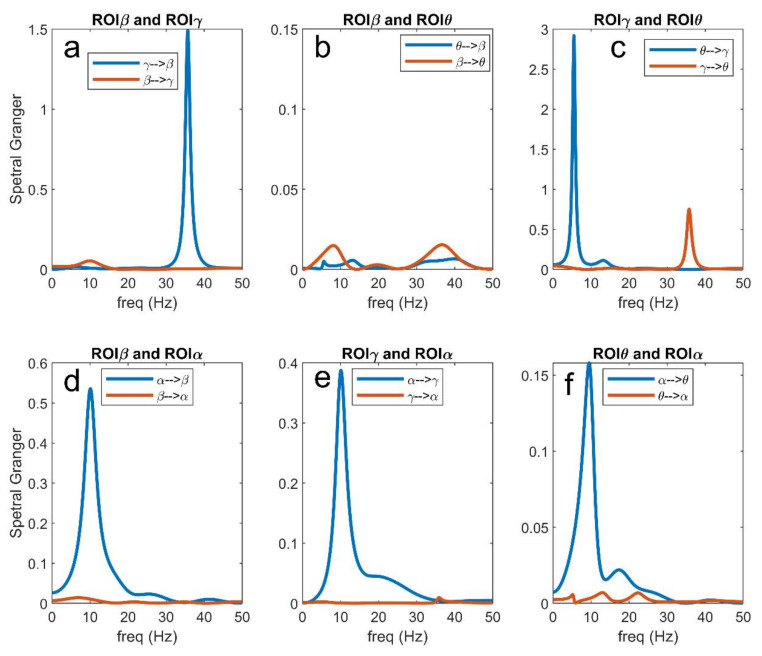
Values of connectivity among the ROIs estimated with the spectral Granger causality, with reference to the network in Figure 2A but with the input to ROIβ reduced from 400 down to 100. The values are plotted as a function of frequency (note the use of different *y*-axes to emphasize the different cases). From the comparison of these panels with those in Figure 11 emerges that: the absence of α rhythm transmitted from ROIβ to ROIγ is evident (**a**); the coupling between ROIθ and ROIβ is negligible (**b**); the transmission γ→θ is significantly reduced, while that θ → γ is increased (**c**); the α rhythm transmission from ROIα to ROIβ (**d**) and from ROIα to ROIγ is increased (**e**); while the α rhythm transmission from ROIα to ROIθ (**f**) is similar.

**Figure 13:**
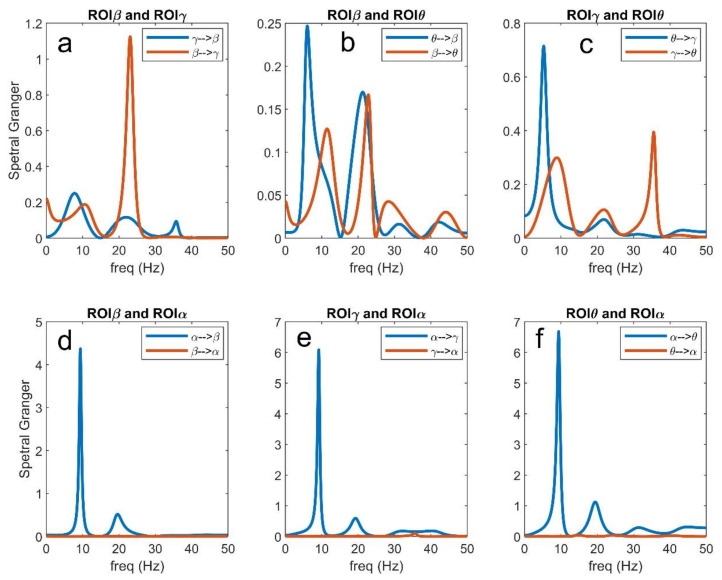
Values of connectivity among the ROIs estimated with the spectral Granger causality, with reference to the network in Figure 2A but with the input to ROIα increased from 200 to 400. The values are plotted as a function of frequency (note the use of different *y*-axes to emphasize the different cases). From the comparison of these panels with those in Figure 11 emerges that: the α rhythm transmitted from ROIα to the other regions becomes almost sinusoidal (high and sharp spectra (**d**–**f**)); the rhythm exchange between ROIβ and ROIγ (**a**) and between ROIθ and ROIγ (**c**) are significantly altered, with the presence of components in the α range; finally, spurious connections appear between ROIβ and ROIθ (**b**), both in the β and α ranges. Comparing this figure with the corresponding bottom panel in Figure 10, the spectral Granger causality provides similar information as the temporal Granger causality, but with emphasis on the frequency bands.

In order to better understand connectivity in the different frequency bands, Figure 11, Figure 12 and Figure 13 illustrate a few examples taken from Figure 7, Figure 8, Figure 9 and Figure 10, using the Granger estimation in the frequency domain. In these figures, each panel represents the connectivity between a couple of regions.

Figure 11 refers to the connectivity network and input values as in Figure 2A. It is evident (Figure 11a) that the region ROIβ transmits a strong information in the β band to region ROIγ, while the information from ROIγ to ROIβ, located at approximately 35 Hz, is less relevant. A strong coupling is also evident between ROIθ and ROIγ in the respective bands (Figure 11c), whereas the coupling between ROIθ and ROIβ is negligible (Figure 11b; also see the different *y*-axes in the figures). Finally, Figure 11d–f illustrate clearly how the α rhythm is transmitted from ROIα to the other regions, without receiving any relevant rhythm back. We observed the stronger transmission γ → θ, as already underlined above. Basically, Granger in the frequency domain produces similar results as Granger in the temporal domain but adds very useful information on rhythms transmission in different bands.

Figure 12 illustrates what is occurring when the input to ROIβ is reduced from 400 to 100 (see also Figure 7). In this condition, ROIβ does not transmit its β rhythm to ROIγ, while the transmission γ → β increases (panel a). Due to a smaller activation of the ROIγ, the rhythm transmitted from ROIγ to ROIθ is also reduced compared with the previous case, while θ → γ increases (panel c). Coupling between ROIθ and ROIβ is still negligible (panel b), but we observed an increased transmission of the α rhythm from ROIα to ROIβ (panel d) and also a small increase in the transmission from ROIα to ROIγ (panel e). Finally, the transmission from ROIα to ROIθ (panel f) is similar as in the previous figure. These results summarize well how an alteration in the input modifies the capacity of a region to transmit its rhythm to others and to receive rhythms from others.

Figure 13 shows the effect of an increase in the input to ROIα from 200 to 400. The dramatic increase in the α rhythm propagated from ROIα to the other three ROIs is evident (panels d–f). In particular, this rhythm becomes almost sinusoidal, as evident by the sharp peak in the spectra. As a consequence, a rhythm becomes relevant everywhere in the net and is also transmitted between other regions. In particular, ROIβ and ROIγ now exhibit a significant transmission in the α band, while ROIγ almost completely loses its capacity to transit the γ oscillation to ROIβ (panel a). The ROIγ is also able to transmit an α rhythm to ROIθ, but the capacity to transmit its intrinsic γ rhythm is drastically reduced compared with Figure 11c. Finally, and more importantly, a spurious connectivity appears in panel B, where the regions ROIβ and ROIθ, not physically connected according to the schema in Figure 2A, apparently exchange information in both α and β bands. This agrees with the spurious connectivity illustrated in Figure 10.

Further exempla concerning the schema in Figure 2B are illustrated in Supplementary Materials part 2 (see Figures S5 and S6).

## 4. Discussion

Rhythmic oscillations in brain activity are known to play a relevant role in many cognitive tasks, including memory, attention, binding and segmentation of perceptual experience, motor actuation [17,18,19,20]. Furthermore, several results suggest that different human cortical regions are characterized by a dominant oscillation, the so-called natural frequency, as demonstrated by single-pulse transcranial magnetic stimulation (s-TMS) [24,26] or by electrocorticogram [48]. In particular, α oscillations (8–12 Hz) dominate over the parieto-occipital cortex, and are also significantly related with attentional modulation [49,50,51]; low β oscillations (13–20 Hz) are evident over the left superior parietal lobule (BA 7) [24] and the dorsolateral prefrontal cortex [26], while faster frequencies (21–50 Hz) occur over the left premotor cortex and anterior areas [24]. Gamma oscillations are also frequently observed in the prefrontal cortex and in the hippocampus, which are associated with the θ rhythm; in particular, γ − θ coupling is related with working memory tasks and spatial memory tasks [19,47]. Moreover, several studies reveal that the local rhythm, evoked by TMS, spreads toward further connected regions [24,31]. All these data suggest that propagation of rhythms among brain regions is a fundamental instrument to realize complex cognitive functions, leading some authors to postulate the existence of a “system of rhythms” subserving cognition [52].

This “system of rhythms”, of course, needs a network of connections, which permits the transmission of oscillations from one region to another, the possible synchronization/desynchronization among neural activities, and the coordinate exchange of reciprocal information. Indeed, the study of brain connectivity is playing a major role in cognitive neuroscience today. A common method to evaluate these connections, from the perspective of brain rhythms, is to apply methods for estimation of functional connectivity to neuroelectrical signals (such as EEG or MEG measurements which encompass a sufficient temporal dynamics). Although the typical analysis of FC in humans occurs through noninvasive methods, there is also the possibility to perform invasive connectivity studies via ECoG—for instance, during a presurgical evaluation of epilepsy (see [11], for a review). In this case, the objective is to quantify the involvement of the different regions in the triggering of the seizure. Actually, our analysis can also be used to analyze relationships between mean field potentials derived from invasive measurements.

Hence, in order to challenge methods for FC estimation, we need a biologically inspired model able to simulate these rhythms to analyze their reciprocal transmission and the effect of nonlinearity, which play dominant roles in brain dynamics.

Unfortunately, most models used in past years for FC analysis are nonoscillating and often make use of linear equations [2,5,6]. Models which make us of spiking neurons are too complex to simulate dynamics of entire brain regions and are suitable for the analysis of neuron cultures in vitro [13]. A few previous studies which investigated FC with neural mass models made use of equations with just two populations (excitatory and inhibitory) or just two interconnected regions [3,14,16]. Hence, we think the present model represents an important advancement in the present literature.

The aim of the present work was to evaluate the relationship between rhythms transmission among ROIs, network connectivity, and methods for FC estimation, laying emphasis on the possibility to detect the strength of the reciprocal connections and, above all, the effect of nonlinear alteration in neural activity. To this end, we used data simulated with a neural mass model of interconnected populations as a ground-truth. As pointed out by Reid et al. [1], the use of abstract model simulations may offer several advantages compared with more sophisticated models—among others, better intuition of the results, reduced computational costs and the capacity to generalize over several physiological conditions.

The signal characteristics exemplified in the frequency and temporal domains (Figure 3 and Figure S2 in Supplementary Materials part 2) indicate that the present model, although extremely simplified, can grasp some important aspects of real neurobiological signals. Indeed, although the present simulations have been performed with constant inputs, constant characteristics of the noise and constant parameters (i.e., in a certain sense, with a *stationary* model), the spike density of pyramidal neurons and mean field potentials (i.e., quantity z_p_ and *v_p_* in the Equations (3) and (4) of the Supplementary Materials part 1) exhibit rapid short-living fluctuations. As underlined in [43,44], not only frequency but also nonsinusoidal waveform shapes may play a relevant role in neurophysiological processes and behavior. However, in real signals, this intermittency in rhythmic activity is probably even more accentuated than in our model, due to the typical nonstationarity of all biological systems (i.e., the inputs and parameters are never constant as in our simulations).

The validity of the model can be further assessed and its limits pointed out by looking at the spectral patterns of the different interconnected ROIs.

(a) The spectrum in ROIα exhibits a clear peak at about 10 Hz, with smaller contributions in the β band. There are several neurophysiological regions that can exhibit a similar pattern. This rhythm may originate from the thalamus [53]. Moreover, the spectrum in this α region is similar to mu rhythms, observed in the sensorimotor cortex (see [54] where a clear peak at about 10 Hz is associated with a smaller component in the β range). Similar spectra can also be seen in occipital regions in a relaxed state (e.g., see [55,56]).
(b) The spectrum in the ROIγ exhibits a very large peak, which is difficult to observe in real EEG signals in the scalp, or also in signals reconstructed on the cortex starting from scalp EEG data (for instance, using algorithms for source reconstruction). There are several possible explanations. First, the γ rhythm can be attenuated by low-pass filtering properties of the tissue; hence, its presence in the scalp is strongly reduced. However, an evident γ peak can be observed in local field potentials during invasive measurements when a population is stimulated (for example, see [57,58]). We think that these rhythms are typical of specific brain regions (for instance, limbic regions such as the hippocampus, or sensory regions when excited by external stimuli and involved in the binding information, or frontal regions involved in working memory). Hence, it is important that a portion of the model produces a clear γ to be transmitted to other regions. It is probable that, in a real brain network composed of multiple regions, the effect of this rhythm may be less evident than in our four-region model. However, its role is extremely important, and we need a ROI contributing to it.
(c) The spectrum in the ROIβ is similar to that observable in motor or premotor regions during a motor task [29,59].
(d) The spectrum in the region ROIθ exhibits approximately a 1/f trend with smaller peaks at high frequencies compared with lower frequencies. This is probably the region more representative of the behavior of many cerebral regions, usually observed with noninvasive EEG.

Of course, even more realistic signals can be built starting from the present model—for instance, using a greater number of ROIs, a different combination of weights, or even real LFP signals as inputs to the regions. Moreover, in future works parameters may be fitted to real EEG signals to further improve model simulation of real cases.

With such a biologically inspired model, we then tested several FC estimation techniques in the presence of rhythm transmission.

A preliminary comparison among the different estimation methods showed that, at least for the particular problem under study (i.e., rhythm transmission), the Granger connectivity performs better than the other techniques. Hence, most of the analysis in this work was performed with this estimation method (in the temporal and frequency domains), while some results obtained with the other methods are shown in the Supplementary Materials part 2. However, it is important to remark that most estimators require the setting of some parameters. We did not perform an exhaustive search of the best parameter combinations for each estimator, since this is well beyond the aim of the present work, and this aspect may be investigated in future studies.

The poor performance obtained with some estimators, such as the phase synchronization index and the delayed correlation (see Figure 4 and Figures S4 and S5 in Supplementary Materials part 2) deserves a comment; this in part contradicts the more encouraging results obtained by us and others in previous works [12,60,61]. In particular, in a recent paper we showed that the delayed correlation is able to grasp the sign of a connection (either excitatory or bisynaptic inhibitory) quite carefully when two ROIs transmit the same rhythm with a feedback connection (see Supplementary Material in Ursino et al.’s work [12]). Conversely, as shown in Supplementary Materials part 2, in the present study we observed that the sign of the delayed correlation is not always related with the nature (excitatory or inhibitory) of the synapses. Similarly, the phase synchronization metrics provide quite poor results in our simulations. We claim that these differences depend on the coupling among oscillations with different frequencies (in the θ, α, β, γ ranges, as actually occurs in the brain), whereas previous studies were especially focused on synchronization of a unique rhythm from one region to another. A different index, involving phase–amplitude coupling [62] may be more appropriate to study connectivity among different rhythms, and may be tested in future work.

However, the main objective of this work was not to compare the performance of different estimators, but rather to critically challenge the concept of FC. Our aim was to assess whether FC can detect not only network topology, but also possible changes in connectivity strength and, above all, how these estimates are affected by nonlinearity in neural dynamics.

We can summarize some interesting indications for MEG/EEG analyses. Figure 5 simulates cases of simultaneous changes in multiple pathways connecting the investigated ROIs, as may occur in several pathological and physiological conditions (for instance, after a stroke, or after training/conditioning paradigms such as multisensory stimulation training or fear conditioning). Our findings indicate that when several pathways change simultaneously, only large variations in a given connection can be detected (for instance, from a high to small value or vice versa; see Figure 5) due to the high SD of the estimates, whereas care must be taken to interpret small connectivity changes, which can be the result of spurious effects. However, a greater reliability of the estimates can be obtained when the results are mediated over large populations of different subjects; in this case, as shown in Figure 5, the average value across many trials can reflect the connectivity in that population quite well. Another interesting point emerges from Figure 6, where only one connection is changed at a time. The results suggest that the FC estimate is quite accurate when only one pathway is changing, while the others maintain a constant level. This may occur in specific stimulation approaches, able to selectively affect only a given neural pathway; in particular, new approaches are emerging (such as cortical–cortical paired associative stimulation via TMS [63]) that can induce plastic changes of a spatial specific and also functionally specific neural pathway. FC estimates can be reliably applied to these approaches when combined with EEG acquisition (and source reconstruction) to derive quantification of the strength changes induced in the specific neural pathway and quantify the relationship between strengthening of the given pathway and behavior.

A further consideration is the case when a connection has a higher value (as high as 30–40) in our random network. In this case, the estimates exhibit a very high variability (mostly evident in the transmission of the β rhythm, see Figure 5, but also in other rhythms). Our interpretation is that, in this condition, the working point of the target population may be drastically altered by the strong incoming connectivity. In other terms, the target population might be silenced, in the case of strong inhibition, or largely excited, in the case of strong excitation, which may trigger nonlinear phenomena, resulting in a completely different capacity to receive or transmit rhythms. This might explain the enormous variability of the estimated connections. Of course, other multivariate aspects (such as the presence of a common source, mixed inputs, or the presence of multiple pathways) can also affect the estimation in the random net. A future paper can evaluate this phenomenon using multivariate algorithms too, such as the multivariate transfer entropy [64] or the partial directed coherence [65].

The previous consideration moves our analysis towards the most important aspect of this study—i.e., the role of nonlinearity (specifically, the population working point) in connectivity estimation.

Although in this study a thorough analysis on the effect of input changes and nonlinearity has been performed using the Granger methods (both in the temporal and frequency domains, Figure 7, Figure 8, Figure 9, Figure 10, Figure 11, Figure 12 and Figure 13, and Figure S3 in Supplementary Materials part 2), similar results have been obtained with all estimators, with only moderate differences (see Supplementary Materials Figures S4 and S5). All metrics agree in that they show that a change in the input to a region causes well evident and repeatable alterations in the “apparent” connectivity—i.e., the estimated functional connectivity. This can be ascribed to a dramatic alteration in the capacity to propagate or receive a rhythm, which occurs despite structural connectivity is unchanged. To investigate this phenomenon, we used quite a simple network inspired by biology, where the α rhythm (occipital/thalamic) inhibits the other regions, and the γ rhythm (fronto-temporal) strongly interacts with the β (motor) and θ (hippocampal) populations. However, similar results can be obtained using different nets too. We tested different excitation/inhibition nets (unpublished simulations) confirming this result (an example, concerning a simple chain of interconnected rhythms is shown in Supplementary Materials part 2, Figures S5 and S6). We also tested a network identical to that shown in Figure 2A, but with excitatory connections only, obtaining similar results.

Despite the presence of clear differences between one region and another, some major common points can be drawn: first, the capacity to generate and transmit a rhythm increases when the population of pyramidal neurons is working in the central linear region of the sigmoidal relationship, but this capacity decreases significantly when the population enters into the (upper or lower) saturation regions; second, in the latter condition a ROI becomes much more affected by the incoming rhythms (i.e., the rhythms arriving from other ROIs which send their synapses into). These aspects are especially evident with reference to the β rhythm due to the smaller amplitude of this oscillation, which makes it strongly dependent on the working point in the sigmoidal relationship but visible to all rhythms and all estimators.

Particularly, results obtained on the α rhythm, by varying the input strength, deserve attention (Figure 10). They suggest that the capacity to propagate α from one region to another dramatically changes if the ROIα is excited, and this is reflected in a stronger presence of the α in the other connected regions. We think that this result agrees with the literature and may shed light on some aspects of the brain α. Indeed, many data in the literature show that the α rhythm can exhibit dramatic changes from one mental state to another (this is especially evident in the occipital cortex, but frontal regions can also exhibit significant changes—for instance, during working memory tests). α is the only rhythm that can either increase or decrease compared with basal conditions [51]. Furthermore, it is a marker of stress, fatigue, and can change dramatically with closed or open eyes. Finally, there is large consensus that α is linked to attention and top-down influences, and an increase in α denotes suppressed activity in regions unessential for the specific task (for references on these aspects, see [66,67,68] as reviews, but also [69,70]). Hence, the fact that α power can strongly change as a function of a top-down mechanism (maybe depending on the thalamus or higher frontal regions) agrees with present knowledge on the role of this rhythm; in this regard, the model may represent a promising instrument for the mechanistic interpretation of this rhythm propagation in various states.

Briefly, although the methods for FC estimation (and, in particular, those based on Granger causality) can be extremely useful to understand how a rhythm is received or transmitted from one ROI to another (see, for instance, the frequency plots in Figure 11, Figure 12 and Figure 13), the values do not always reflect the true structural connectivity within the network, being strongly influenced by the particular working conditions in which the net is actually operating. In particular, the presence of a strong rhythm transmitted from a presynaptic region to a postsynaptic region can be due not only to a strong connectivity but can reflect the particular conditions of the receiving ROI, especially when the latter is scarcely excited by other external inputs and becomes more prone to the incoming influences. Similarly, the presence of a poor rhythmic transmission does not necessarily reflect the absence of a causal link but may signify that the source region is scarcely active at that moment, or that the source region reached an upper saturation, which significantly depresses its rhythmic variability. In other words, estimators reflect how much information is actually transmitted in a particular frequency domain, and in a particular operational mode, but this does not always correspond to structural connectivity.

This argument has two main implications for connectivity experimental studies. First, caution must be taken when interpreting the comparison between connectivity networks obtained during different tasks (for instance, in resting conditions and during task execution), since a task may alter the working conditions of some regions. Conversely, most studies making use of FC estimation techniques directly compare the networks obtained during different tasks and try to interpret these differences merely in terms of a change in the underlying connectivity. Indeed, a frequent conclusion in the literature is that a task dramatically alters the structure of the connectome. In our opinion, this conclusion may be questionable if the connectivity network is believed as a real physical structure—i.e., if we are looking for physical synapses linking the regions. Moreover, it is worth noting that this misinterpretation (i.e., assuming any change in the transmitted information as a true change in physical connectivity) is frequently made by using methods for effective connectivity estimation (i.e., methods that consider an underlying model). This is particularly true when these methods make use of a linear model to infer causal parameters from data [5,6,7], since these models neglect nonlinearity.

Of course, we do not exclude the possibility that some physical connections can change quite rapidly, as a function of the particular task (for instance, reflecting receptor binding by neurotransmitters), but we suspect that at least part of task-dependent connectivity changes reflects nonlinear phenomena, as illustrated in the present study, rather than a structural connectivity alteration.

## 5. Conclusions

In conclusion, our study critically examines the concept of functional connectivity, using a biologically inspired model as ground-truth, which generates nonsinusoidal oscillations in different frequency bands. An important aspect of the study is the emphasis on rhythm propagation and on its dependence on nonlinear phenomena typical of neural systems.

As an innovative emerging concept, we wish to underline that results obtained through FC estimators, reflecting information exchange among ROIs, can provide evidence not only on the network connectivity, as usually carried out in the literature, but also on how a region may be activated or silenced during the given task as a function of other external influences. This may lead to a more complete and innovative analysis of brain functioning. Since different tasks determine different working conditions in neural populations, a more exhaustive analysis of brain functioning should move from a linear to a nonlinear perspective (although the latter is mathematically more complex), and from network connectivity metrics to metrics which incorporate connections, inputs and working conditions altogether.

## Figures and Tables

**Table 1:** The different FC estimators.

FC Estimator	AUC
Correlation	0.6987
Delayed Correlation	0.7580
Phase Synchronization	0.7100
Lagged Coherence	0.7465
Coherence	0.7673
Transfer Entropy	0.7753
Temporal Granger	0.8787
Spectral Granger	0.8759

## Data Availability

The neural mass model and the functional connectivity estimators were implemented in Matlab. Matlab codes are available in the repository ModelDB (A Neural Mass Model to evaluate the relationship between Brain Rhythms and Functional Connectivity; Ricci et al.) at http://modeldb.yale.edu/266980 (accessed on 12 April 2021).

## Supplementary Materials

The following are available online at https://www.mdpi.com/article/10.3390/brainsci11040487/s1, Supplementary Materials part 1: Quantitative model description and parameter values: Table S1: Part I—Parameters assumed fixed for the four population and Part II—Parameters values used for the four populations, Supplementary Materials part 2: Further Simulation Results: Figure S1: Power density spectrum of the noise, filtered by the dynamics of the glutamatergic synapses and multiplied by the constant C*_pe_*, Figure S2: Spectrograms of the potential for the pyramidal population in the four different ROIs simulated with the connectivity, Figure S3: Relationship between the connectivity estimated with the Temporal Granger estimator and the true connectivity, using the data obtained from 100 randomly generated networks, Figure S4: Values of connectivity among the ROIs estimated with the eight different FC estimators, with reference to the network in Figure 2A, Figure S5: Values of connectivity among the ROIs estimated with the eight different FC estimators, with reference to the network in Figure 2B (i.e., a circular connections), Figure S6: Values of connectivity among the ROIs estimated with the Temporal Granger Causality, with reference to the network in Figure 2B.
